# Current and Future Nano-Carrier-Based Approaches in the Treatment of Alzheimer’s Disease

**DOI:** 10.3390/brainsci13020213

**Published:** 2023-01-27

**Authors:** Astik Kumar, Sachithra Thazhathuveedu Sudevan, Aathira Sujathan Nair, Ashutosh Kumar Singh, Sunil Kumar, Jobin Jose, Tapan Behl, Sabitha Mangalathillam, Bijo Mathew, Hoon Kim

**Affiliations:** 1 Department of Pharmaceutics, Amrita School of Pharmacy, AIMS Health Sciences Campus, Amrita Vishwa Vidyapeetham, Kochi 682041, India; 2 Department of Pharmaceutical Chemistry, Amrita School of Pharmacy, AIMS Health Sciences Campus, Amrita Vishwa Vidyapeetham, Kochi 682041, India; 3 Department of Pharmaceutics, NGSM Institute of Pharmaceutical Science, NITTE Deemed to be University, Mangalore 575018, India; 4 School of Health Science and Technology, University of Petroleum and Energy Studies, Dehradun 248007, India; 5 Department of Pharmacy, and Research Institute of Life Pharmaceutical Sciences, Sunchon National University, Suncheon 57922, Republic of Korea

**Keywords:** neurodegenerative disorders, blood–brain barrier, Alzheimer’s disease, Parkinson’s disease, amyloid beta, liposome, nanosomes, cubosomes

## Abstract

It is a very alarming situation for the globe because 55 million humans are estimated to be affected by Alzheimer’s disease (AD) worldwide, and still it is increasing at the rapid speed of 10 million cases per year worldwide. This is an urgent reminder for better research and treatment due to the unavailability of a permanent medication for neurodegenerative disorders like AD. The lack of drugs for neurodegenerative disorder treatment is due to the complexity of the structure of the brain, mainly due to blood–brain barrier, because blood–brain drug molecules must enter the brain compartment. There are several novel and conventional formulation approaches that can be employed for the transportation of drug molecules to the target site in the brain, such as oral, intravenous, gene delivery, surgically implanted intraventricular catheter, nasal and liposomal hydrogels, and repurposing old drugs. A drug’s lipophilicity influences metabolic activity in addition to membrane permeability because lipophilic substances have a higher affinity for metabolic enzymes. As a result, the higher a drug’s lipophilicity is, the higher its permeability and metabolic clearance. AD is currently incurable, and the medicines available merely cure the symptoms or slow the illness’s progression. In the next 20 years, the World Health Organization (WHO) predicts that neurodegenerative illnesses affecting motor function will become the second-leading cause of mortality. The current article provides a brief overview of recent advances in brain drug delivery for AD therapy.

## 1. Introduction

Neurodegenerative disorders are alterations of the neuronal networks, which cause neuropathological conditions in which the aggregation of protein takes place due to excess amounts of copper and iron that lead to oxidative damage in the central nervous system (CNS) [1]. It is approximated that there are more than 276 million people affected by neurodegenerative disorders and this is the second leading cause of death by a type of disorder; among these people, currently, 55 million people are affected by Alzheimer’s disease (AD) alone [2,3]. AD mostly affects elderly people through loss of memory (dementia), which leads to behavioral changes and eventually causes difficulties in performing their daily activities [4]. One other neurodegenerative disorder is Parkinson’s disease (PD); tremors, muscle rigidity, and slowness of movement are prominent signs of this condition, which is caused by the death of certain neurons that create dopamine. All of these are referred to as motor symptoms because they are all related to movement. Many PD patients also have additional issues in addition to mobility, such as discomfort, pain and anxiety. According to a study, by 2040 approximately >13 million people will be affected by PD [3]. There are several marketed drugs and approaches available to treat AD and PD, but they act only for a short period of time or only minimize the symptomatic effects, such as confusion, hallucination, dementia, and loss of appetite.

**Figure 1:**
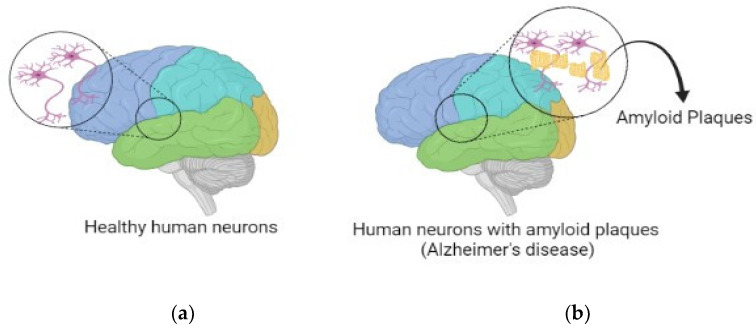
Human brains having (**a**) healthy neurons (**b**) amyloid plaques in neurons.

According to the 2016 World Alzheimer’s Report, approximately 47 million people worldwide are suffering from AD or associated dementia. After 20 years, the number will double, with 130 million people being affected by AD by 2050. According to the report, 58% of patients live in low-income countries, so numbers are expected to rise to 68% by 2030 because proper AD medicine is lacking in this decade [5]. The most common cause for neurodegeneration in the old age is AD [4]. Impaired neuronal signaling is the effect of AD that causes a slow and progressive decline in cognitive and behavioral function [6]. It is characterized by memory loss that interferes with daily living, and thus, it takes longer to conduct everyday routine duties, accompanied with repetition of questions or forgetting newly acquired knowledge, problem-solving difficulties, wandering and being disoriented, losing or misplacing items in unusual settings, changes in mood and personality, and an increased degree of anxiety and/or hostility. Though the proper aspects of the etiology of this disease are still under investigation, the major focus is on the ‘cholinergic hypothesis’ [4]. The cholinergic hypothesis links with the dysfunction of cholinergic signaling in the hippocampus and cerebral cortex [6]. Another hypothesis regarding the characteristics of AD is the improper regulation of amyloid beta (Aβ), which combines to produce amyloid plaques. When a much bigger protein known as the amyloid is broken down, the Aβ is formed. That is, APP comprising of 771 amino acids is broken down by two enzymes, i.e., beta-secretase and gamma-secretase, and results in Aβ fragments consisting of 38, 40, or 42 amino acids. Since the Aβ consisting of 42 amino acid is chemically “stickier” than the other fragment lengths, plaque formation is more likely, and it forms the accumulation of senile plaques in large quantities [7,8] (Figure 1). On the other hand, the possibility of using of human chaperones to target Aβ deposits, as well as the potential of application of amyloid-targeting proteins isolated from other non-human organisms, can be considered in AD treatment; this is based on the concept that the similarity of amyloid folds on a molecular level between different organisms gives hope that the usage of anti-amyloid and anti-prion proteins and systems from these organisms potentially may be beneficial for the treatment of human amyloid diseases [9,10].

Neurons pass the information signals through the cytoskeleton, which is made up of microtubules, and these microtubules are stabilized with the help of tau proteins. In the case of AD, these proteins become defective and detach from these tubules. This leads to the distortion of the skeleton of the neuron and the shape of the neuron changes. These deformed tau proteins cause the formation of neurofibrillary tangles and result in AD. The human body has two cholinesterase enzymes: acetylcholinesterase (AChE) and butyrylcholinesterase (BuChE). Inhibiting these enzymes with cholinesterase inhibitors (ChEIs) has been shown to be useful in treating AD, and these medicines are now regarded to be the most efficacious treatments for the pharmacological treatment of mild-to-moderate AD. The use of ChEIs for AD therapy is based on the notion that cognitive impairment in AD patients is caused by a deficiency or loss of cholinergic neurons in the basal forebrain. ChEIs are useful in this situation because they are hypothesized to prolong the half-life of acetylcholine by inhibiting AChE and BuChE. ChEIs, such as donepezil, galantamine, and rivastigmine, have been utilized as first-line pharmacotherapy to treat mild-to-moderate AD [4].

## 2. Pharmacotherapy Approaches in AD Treatment

For the suppression of AD symptoms, there are several medications on the market and in clinical studies. The primary objective of these medications, both marketed and in clinical trials, is to identify the true pathophysiology of the illness and inhibit it. Several delivery routes are now under development for this purpose, and many more are accessible. Below are some of the pharmaceutical treatments for AD.

### 2.1. Drug Repurposing

Drug repurposing, also known as drug repositioning or drug reprofiling, is the process of finding new uses for a medication that has previously been established and is now being used to treat issues other than AD [11]. This process for the discovery of a drug is rapid and economical in comparison to other approaches. The idea of repurposing drugs is aligned with the concept of polypharmacology. Because of its accessibility in the market, it shows significant results for the pharmacokinetics, toxicological and safety data [12]. Currently, this strategy is working successfully for various issues, such as PD, cancer, erectile dysfunction, and cardiovascular disease [13,14]. According to Corbett et al., nitrendipine (antianginal action) can reduce dementia risk in elderly people over the age of 55 by 56%. In addition, the researchers hypothesized that “calcium channel blockers might minimize the likelihood of accumulation of Aβ proteins if the larger dose is provided” based on their in vitro investigation [14].

We reviewed publications to find chemicals that are USFDA-approved for the treatment of conditions other than Alzheimer’s, but have an effect on AD modification based on clinical data because existing medications for AD only help in symptom alleviation and do not help in disease reversal. It is feasible to take into account amyloid clearance, tau pathology, and anti-inflammatory mechanisms while managing the condition. Drug selection is based on in vitro research, animal models, and human observational studies [10].

#### 2.1.1. Thalidomide

This inhibits tumor necrosis factor-alpha (TNFα), and it also helps in the reduction in tumor growth through anti-inflammation and anti-angiogenesis. Through the suppression of TNFα, neuronal damage is reduced and cognitive function is restored. It then regulates the development of senile plaques by lowering glial cell activation and lowering the Aβ burden [15]. Thalidomide is, therefore, effective in treating dementia.

#### 2.1.2. Bexarotene

Bexarotene received USFDA clearance for the treatment of cutaneous T-cell lymphoma in 1999 [15]. “The retinoid X receptor agonist bexarotene enhances the quick clearance of ApoE-dependent Aβ protein molecules, lowering Aβ plaque in the brain and restoring social and cognitive behavior in the individuals,” according to Landreth’s group research [16]. It demonstrates a beneficial impact on AD.

#### 2.1.3. PD 1 Blocker 

Programmed cell death 1 (PD1) blockers, such as Keytruda, Opdivo, and others, are anti-cancer medications that strengthen the immune system and help the body fight off malignant cells. This PD 1 blocker exhibits a decrease in cerebral A protein owing to its immune response in the cerebral cortex area and the dentate gyrus region of the hippocampus, which are the two primary sites of amyloid beta protein deposition, according to a study of two mice models [17].

#### 2.1.4. Anti-Microbial 

Anti-microbials, such as azithromycin and erythromycin, were tested by checking the expression level of APP (amyloid precursor proteins). In the 5′- untranslated region of the APP gene, a nuclear factor binding protein (DABP) was recognized. Disposing of DABP will minimize the overall expression from the amyloid precursor protein promoter [18]. Due to this minimization of the APP promoter, antimicrobials such as erythromycin reduce Aβ protein at the cerebral level. Erythromycin can induce the expression of a 7-kDa APP C-terminal fragment, which leads to the activation of neuroprotective genes [10].

#### 2.1.5. Clioquinol 

In the transgenic mouse model of AD, Aβ levels were decreased to an excessive level while working memory was improved. Clioquinol has the ability to cross the BBB and bind to copper (II) and zinc (II), which results in the buildup of Aβ and promotes the development of illness. In the TgCRND8 mouse model of AD, clioquinol application results in a favorable response [19].

#### 2.1.6. Anti-Diabetic

Agents rosiglitazone and pioglitazone act as agonists of the peroxisome proliferator-activated receptor γ to treat diabetes mellitus II. According to the in vitro evaluation, Rosiglitazone displays a partial neuroprotective effect, while with the combination of insulin it shows good neuroprotective effects [20]. Rosiglitazone reduces the accumulation of the Aβ protein and inflammation and improves cognitive decline [21].

Many studies have reported that diabetes mellitus (type II) can develop into AD. The insulin-based approach is a novel approach adopted to reduce cognitive decline and damage to nerve cells. To help those with AD, several strategies, including intravenous insulin infusion and intranasal insulin delivery, are being used [10]. Table 1 lists some of the key characteristics and structures of repurposed medications.

### 2.2. Oral Administration

#### 2.2.1. Conventional Oral-Based Delivery

ChEIs and N-methyl-D-aspartate [NMDA] receptor antagonists are currently the two approaches being used to alleviate the symptoms of AD [30]. ChEIs are the first-line treatment option for oral-based AD treatment because it slows down acetylcholine deterioration in synaptic clefts and compensates for its deficiency [31]. Tacrine’s usage is confined to mild and severe forms of AD since it has a hepatotoxic side effect [32]. In 2003, the USFDA approved memantine, an NMDA receptor agonist, for use in patients with mild-to-severe AD problems [33].

##### Donepezil

In 1996, the US approved donepezil as an irreversible, non-competitive and selective AChE inhibitor for the treatment of dementia; however, it also causes gastrointestinal and sleep disorders without affecting peripheral AChE [34].

##### Rivastigmine

Rivastigmine, which functions as a dual inhibitor for the inhibition of AChE and BuChE, was licensed by the EU in 1998 as a candidate for the treatment of mild-to-moderate types of dementia. The USFDA later approved this medicine in 2000. Gastrointestinal issues are the major side effect linked to the use of rivastigmine [35].

##### Galantamine

Galantamine exhibits a protective effect against mild-to-moderate AD, much like donepezil and rivastigmine. Galantamine was the first major ChE inhibitor to be made accessible in its generic form while being the last of the three major ChE inhibitors to obtain FDA clearance [36].

##### Memantine

Memantine received USFDA clearance in October 2003 to treat moderate-to-severe stages of AD. Because of its long plasma half-life (60 to 80 h), a dosage plan of 20 mg/day is advised [37]. The properties of these drugs are listed in Table 2.

### 2.3. Oral Novel Drug Delivery

Orally ingestible medications, including rivastigmine, galantamine, donepezil, and memantine, as well as transdermal galantamine, are the mainstays of anti-dementia therapy. However, dementia renders it difficult for the patient to take medication at the appropriate dose interval; therefore, to circumvent this restriction, new methods of administering the dose to the CNS must be devised, together with considerations for cost and convenience of administration. Alternatives to the traditional dose form include oral dissolving or sublingual formulations, extended release (ER), transdermal, pulmonary, intranasal, short/long-acting intramuscular, and, more recently, innovative medication therapies, including nanotechnology-based drug delivery. Each delivery method has pros and cons in terms of cost, efficiency, delivery method, and patient preferences. As a result, an unconventional or novel dosage form has been developed for dementia patients who need fewer frequent doses or an extended-release formulation [38,39].

### 2.4. Oral Traditional Dosage Form 

Traditional drug delivery systems (DDSs) or drug-integrated formulations are available in bulk, micro- or nanoforms, such as tablets, suspensions, powders, capsules, sprays, and so on. Administering extended-release drugs through the oral delivery dosage form is an unconventional approach for the delivery of drugs, and it is beneficial for dementia patients because it releases the drug in a controlled manner so it reduces the frequency of dose administration to just once a day. There are several approaches for designing the extended-release oral formulation for oral delivery, such as diffusion and/or erosion-controlled polymer and the osmotically controlled polymer matrix, which is designed to release the drug at a fixed rate. The advantages of innovative dosage forms over traditional ones include reduced drug administration frequency, consistent drug levels, sustained drug effects, smooth plasma concentration levels associated with adverse effects, and improved patient comfort and compliance. There are specific drawbacks to innovative methods, such as the fact that novel dosage forms are costlier than traditional dosage forms and also if the medicine is crushed, fractured, or chewed, it is prevented from dispensing at a consistent set rate.

As innovative drug delivery methods, medications including donepezil, galantamine, physostigmine, and memantine are being developed [40].

#### 2.4.1. Donepezil 

USFDA approved donepezil hydrochloride in 2010 as a sustained-release (SR) film-coated tablet meant for once-a-day administration via an oral route, which contains 23 mg of donepezil hydrochloride sufficient for mild-to-severe AD. Research that involved 1500 participants over 219 locations globally found that subjects taking 23 mg/day saw significant cognitive gains in addition to functional benefits even when they had severe AD [41]. One of donepezil hydrochloride’s main drawbacks is its bitter taste, hence a novel microsphere-loaded tablet formulation has recently been developed to improve patient compliance [42].

#### 2.4.2. Galantamine

Galantamine was approved in 2004 and made accessible for usage in the US as an opaque hard gelatin extended-release capsule containing salt hydrobromide. It comes in three colors, white, pink, and caramel for 8, 16, and 24 mg, respectively, depending on the concentration [43].

Each capsule has a 75% dose in a controlled-release form and a 25% dose in an immediate release form. A total of 24 mg of extended-release capsules when administered orally in fasting conditions show the bioequivalent effect of the immediate-release tablets when administered twice a day with the dose of 12 mg concerning C_max_ (maximum serum concentration, a medication reaching a particular bodily test compartment following the administration of the medication but prior to the administration of a second dosage) and AUC_24hr_ (area under curve, a measure of how a drug’s blood plasma concentration changes over time) [44]. Gastrointestinal issues are evident when at least 5% of both galantamine ER and IR are taken. Another method of delivering galantamine hydrobromide in the oral cavity is by the use of a medical device called IntelliDrug, a controlled drug delivery oral device intended for transbuccal administration [45].

#### 2.4.3. Physostigmine

Physostigmine has the beneficial property of being absorbed by the gastrointestinal tract and undergoing presystemic metabolism, but its oral administration is affected by lower bioavailability and low-dose frequency, which leads to poor patient compliance [46]. Bolourchian et al. recommended the development of tablets with the appropriate chemical and physical qualities for the sublingual distribution of physostigmine salicylate in order to minimize these issues [47]. Additional in vivo research is still being conducted.

#### 2.4.4. Memantine

Memantine obtained approval in June 2010 from the USFDA for moderate-to-severe dementia treatment as an extended-release capsule containing 28 mg of the drug (Namenda XR^®^). Each capsule contains beads responsible for the extended release of memantine hydrochloride at the preprogrammed amount. Initially, the treatment begins with a dose of 7 mg/day and is gradually hiked up (7 mg/day increment) until the target dose is achieved. For a typical patient, the target dose is 28 mg/day, while those patients who have complications of severe renal impairment have a target dose of 14 mg/day [48].

### 2.5. Transdermal Drug Delivery

Oral administration has several limitations, including first-pass metabolism, limited effectiveness, and low bioavailability, which make it difficult to treat AD-like conditions. An alternative method is transdermal administration, which allows the sustained diffusion of active ingredients, avoids first-pass metabolism, and reduces systemic adverse responses. The FDA approved five drugs for AD. Rivastigmine as a cholinesterase inhibitor, and tacrine, donepezil, galantamine and memantine as antagonists of the NMDA receptor [49]. This improves patient compliance and enables the drugs to be used for a prolonged period of time.

#### 2.5.1. Cholinesterase Inhibitors 

According to the oldest theory, a lack of acetylcholine is the cause of AD. Transdermal delivery might make it easier to distribute medications via the skin smoothly and consistently and provide long-term effectiveness with relatively modest doses [50]. Drugs that are used as cholinesterase inhibitors are discussed below.

##### Physostigmine

The first anticholinesterase medicine to be applied transdermally for AD treatment was physostigmine. There are certain issues with the oral and intravenous administration of physostigmine that can be minimized with a patch, including reduced bioavailability, a narrow therapeutic window, high first-pass metabolism, and poor user compliance [46]. A patch consisting of physostigmine in the enhancer carrier propionic acid and a vinyl acetate/ethylene copolymer membrane with an aluminum foil cover on one side and an adhesive on the other were developed by Levy et al. in 1986 [51]. As a result, the Lohmann Therapie-System GmbH created a physostigmine transdermal system with 30 mg active molecules and a surface area of 30.2 cm. The absorption was stable from 8 to 22 h after administration, according to the in vitro release. The test on humans confirmed prior findings, such as the therapeutic range remaining stable for 18 h following a single dose [52]. For better penetration, a transdermal drug delivery system for physostigmine was developed using a non-ion surfactant polyethylene glycol copolymer.

##### Tacrine

Tacrine was one of the experimental medications used in the early phases of transdermal therapeutic strategies against AD. Tacrine has a reversible cholinesterase inhibitor and a lipophilic and weak base characteristic that made passive diffusion challenging, so ion-exchange fibers and iontophoresis were used to transfer the materials [53]. Electrorepulsion and electroosmosis drove the medications into the skin since they had the same polarity as the electrode. Iontophoresis is influenced by a variety of parameters, including drug concentration, molecular size, and donor buffer strength, as well as electrical characteristics such as current density and mode [54]. The results demonstrated that the distribution of active substances in humans was successfully regulated with the use of an iontophoresis system, reaching a steady state of around 14.9 ± 2.6 ng/mL till the device was shut down [55]. Despite its cognitive advantages, in 2013 due to hepatotoxicity-related side effects, it was pulled out from the market [56].

##### Rivastigmine

In the 1970s, the rivastigmine transdermal patches were developed and later in 1979 they obtained the approval from the FDA [57]. The primary generation of patches was a basic reservoir that consisted of an alcohol-based medication and a solution-soaked adhesive edge that was made up of a plastic sheet; nevertheless, this device produced skin discomfort. The next step in the creation of the matrix patch was to overcome this flaw. This method’s four layers help the medication release consistently, reducing skin problems and increasing the time of adherence [58].

**Figure 2:**
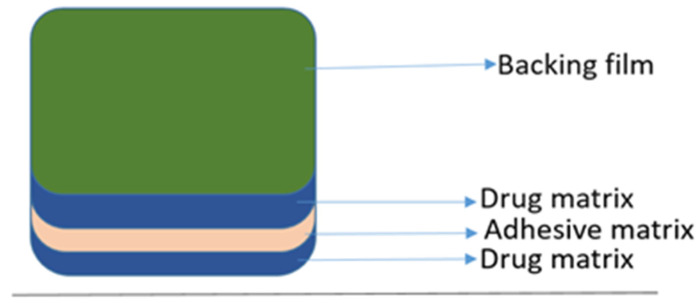
Rivastigmine patch.

This patch contains the drug, antioxidants, and the polymer matrix along with a silicon matrix adhesive in a single layer. Because of this initiative, the thickness and surface of the patch were diminished, which created a drug administration that followed patient compliances, as shown in Figure 2. This patch was available in three different sizes in the market (5 cm^2^, 10 cm^2^, and 15 cm^2^) and their flow rate per 24 h was directly proportional to its size (4.6 mg, 9.5 mg, and 13.3 mg, respectively) [56].

During the treatment with this patch, skin irritation was noted, leading to the concept that employing sonophoresis at a low frequency might increase this utility. An ultrasound produced localized transport routes that allowed the medication to penetrate the skin [59]. Due to the structural flexibility of mechanical and chemical manipulation, chitosan, an N-deacetylated derivative of chitin, is the new drug reservoir or drug carrier for release at a controlled rate [60].

##### Donepezil

In late 2008, the transdermal application of donepezil’s prescription dosage form underwent evaluation. It is the most effective cholinesterase inhibitor because of the enzyme’s high potency and specificity in the CNS [61]. Valia et al. suggested two kinds of patches: drug-matrix-in-adhesive patches and drug-reservoir-in-adhesive patches. The second version resulted in a quicker inflow of functional material because of the migration of medication from the reservoir into and via the adhesive layer. These strategies permitted regulated release by correcting the patch’s active surface, which comes into direct contact with the skin [62]. Permeation enhancers and solvents, such as fatty acids, may aid in the diffusion of donepezil into the bloodstream through the stratum corneum. For the penetration test, an excised hairless mouse model was employed, where it was observed that formulations including water, ethyl alcohol, and isopropyl alcohol operating as co-solvents had elevated the fluxes of permeation [63]. Iontophoresis technology was used to penetrate the skin with a patch containing a gel formulation of donepezil. The pharmacokinetic features of the iontophoresis application groups demonstrated a significant increase in dosage delivered per day, T_max_ and C_max,_ when compared to the intravenous route. This is an appealing pharmacological approach for treating AD [64].

##### Phenserine

The disadvantages of phenserine are the same as those of other anticholinesterase drugs, such as digestion, metabolism, and first-pass hepatic absorption. To tackle this challenge, phenserine was produced as an ointment formulation and examined in vitro and in vivo. The in vivo results demonstrated that phenserine induced efficacy in both the brain and plasma, with 30% inhibition and 60% inhibition after 8 h, respectively, displaying enhanced cognitive performance in an animal model [65].

##### Galantamine

Galantamine shows dual activity by inhibiting the cholinergic system while also modulating allosterically nicotinic acetylcholine receptors. FDA approved this drug for oral administration for commercialization [66]. Galantamine was developed as a drug-in-adhesive transdermal patch to provide a new treatment alternative for Alzheimer-affected patients. The effects of formulation elements such as pressure-sensitive adhesives, enhancers, and the concentration of medicine were studied [67]. The ideal patch comprises 8% galantamine and 3% acid oleic in DT-2510 (a –OH group functional pressure-sensitive adhesive), and has a great bioavailability of over 80%, with a stable amount of active component over a long period of time; 24 h in a rabbit model animal study. Because of the gel’s acceptable properties, a transdermal technique for anti-AD was demonstrated. As a result, a gel reservoir formulation of 0.89%^w^/_w_ carbopol, 1.16%^w^/_w_ triethanolamine, and 4.19%^w^/_w_ galantamine was developed. This patch had a substance extraction of 16.93 mg/cm and a true penetration flux of 2.32 mg/h/cm after 8 h, resulting in high drug concentration and a regulated drug release design for the transdermal administration of galantamine [68].

#### 2.5.2. Noncompetitive N-Methyl-D-Aspartate

##### Memantine

It has been assumed that increased NMDA receptor activity was a potential factor in cholinergic cell degeneration. Memantine inhibits the activity of NMDA receptors by blocking them during the prolonged release of low glutamate concentrations. This active chemical has recently been produced in a transdermal dosage form as an alternate technique for the treatment of AD [69]. Del Rio-Sancho et al. explored elements that could enhance memantine’s permeability through the epidermis. Pretreatment with decenoic acid, oleic acid or laurocapram increased memantine transdermal flow by a statistically significant amount (*p* = 0.05), with flux values of 24.5 ± 3.2, 22.6 ± 2.3, and 38.4 ± 4.7 µg/cm^2^ h, respectively. The highest flux value was reported after R-(+)-limonene was used. Apart from that, iontophoresis is the most commonly used physical enhancer, which has been demonstrated to be significantly more successful than other chemical enhancers tested, raising memantine flow 22.3 times when compared to passive diffusion [70]. The memantine patch was assessed in rats for pharmacokinetic parameters. The AUC value of the transdermal patch was 4.3 times greater than those of oral dosages in single doses; however, the C_max_ was equivalent when compared to the oral route. Furthermore, the repeated dosage trial revealed that the AUC_0-24_ of the patch application matched the AUC_0-12_ of the oral approach. Furthermore, compared to the oral formulation, the memantine patch formulation had reduced inter-individual variability, lower accumulation and inter-individual variability [37]. This study proved the feasibility of administering memantine topically to treat AD.

### 2.6. Neurological Preservative

The latest advancement in AD involves targeting the brain’s regenerative neurogenic ability as a potential alternative treatment to avert neurodegeneration and preserve neuro-regenerative function.

#### Allopregnanolone

The impact of allopregnanolone in promoting neurogenesis in the hippocampus and maintaining the normal function of memory after the onset of AD pathology was studied using a triple transgenic AD mouse [71]. Despite having features that make it ideal for aiming at the bases of the brain, such as a low number of hydrogen bond donors, low molecular weight and receptors, this active chemical is inconveniently produced in an aqueous form. As a result, the transdermal use of this molecule in AD treatment would be a unique approach [72]. A gel solution containing allopregnanolone was tested in pharmacokinetic animal models. In a rabbit study, C_max_ was 9.6 ng/mL after 15 min of injection, and after 8 h, the compound’s intravascular allopregnanolone concentration was lower than its brain concentration. Surprisingly, allopregnanolone levels lasted in the animal’s brain for 24 h during the examination, resulting in a 36 percent higher neurological system exposure with transdermal treatment than with IV treatment [73]. 

### 2.7. Nanotechnology

**Figure 3:**
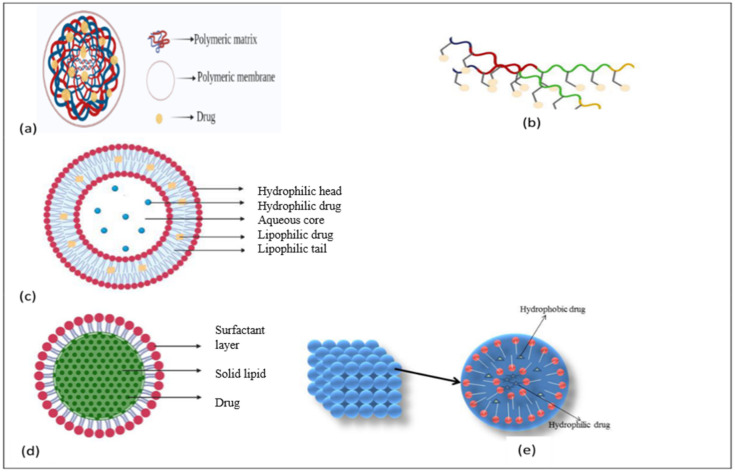
Nanoparticles for AD treatment: (**a**) Polymeric nanoparticle; (**b**) Aptamer; (**c**) Liposomes; (**d**) Solid lipid nanoparticle; and (**e**) Cubosomes.

Nanotechnology is advancing the diagnosis of AD. The development of cerebrospinal fluid biomarkers for illness has received a lot of attention in recent decades. Tau protein, amyloid precursor protein (APP), 42-amino-acid type of β-amyloid (Aβ_42_) and amyloid-derived diffusible ligands were among the diagnostic targets (ADDL). ADDL levels in the cerebrospinal fluid have been linked to dementia. The application of ADDL-specific monoclonal antibodies in the bio-barcode amplification, an ultrasensitive nanoparticle-based protein detection technology, makes it easier to assay these proteins. The bio-barcode amplification uses nanoparticles as DNA carriers to enhance detection sensitivity [74]. Another recent breakthrough is the introduction of gold nanoparticles. The Aβ portion of an antibody was attached to gold nanoparticles that bind to the Aβ protein. The resulting immunocomplexes were identified using scanning tunneling microscopy at concentrations as little as 1 fg/mL. Another application of gold nanoparticles is the construction of multispot-localized surface plasmon resonance immunochips that can track tau at 10 pg/mL [75]. These two technologies will help doctors diagnose AD more precisely. The nanoparticles discussed are mentioned in Figure 3.

#### 2.7.1. Polymeric Nanoparticles

The polymeric nanoparticle (PNP) system in this work is made up of a PEGylated PLGA polymeric matrix that has been formulated in an antioxidant environment with the addition of ascorbic acid. Both ascorbic acid and nanoencapsulation safeguard EGCG’s chemical composition and shield it from in vivo clearance, increasing its bioavailability and activity. The PNP system in this work is made up of a PEGylated PLGA polymeric matrix that has been formulated in an antioxidant environment with the addition of ascorbic acid. Ascorbic acid and nanoencapsulation both safeguard EGCG’s chemical composition and protect it from in vivo clearance, increasing its bioavailability and activity. This PNP enhances neural cell viability and improves cognition and memory deficiencies in treated mice without causing any harmful effects in different organs [76].

Huo et al. developed a curcumin/selenium combo carrier intending to minimize Aβ aggregation. This nanocarrier binds Aβ oligomers selectively, opening up new pathways for the targeted medication [77]. For more effective targeting, Vilella and colleagues formulated a zinc-loaded polymeric nanocarrier associated with the coating of peptide, which is responsible for BBB penetration [78]. The study demonstrated that zinc-loaded PLGA NPs reduced the amount of Aβ plaque and the neuroinflammatory cytokines in transgenic AD mice without altering their behavior.

#### 2.7.2. Aptamers

Aptamers are RNA or single-stranded DNA oligonucleotides, ranging from 20 to 60 nucleotides, similar to antibodies, which harness the ability of nucleic acids. They show less toxicity or immunogenicity along with high affinity and specificity and a long shelf life. Nanomaterials with a microscopic particle size, huge surface area, excellent absorption, and little immunogenicity are promising candidates for nanomedicine due to their physicochemical features. Aptamers with easy chemical alteration at both the 3’ and 5’ ends, together with excellent selectivity and target affinity, provide significant structural benefits in medical imaging and tagging, notably in approaches such as positron emission tomography (PET) and magnetic resonance imaging (MRI) [79].

Aptamers against Aβ will help in the initial diagnosis and treatment of AD because Aβ is the most widespread biomarker for AD. The identification of Aβ and the prevention of Aβ aggregation are the main goals of aptamers.

##### RNA Aptamers

The RNA aptamer is the first aptamer against Aβ. In 2002, Ylera et al. employed the affinity column approach to screen RNA aptamers using the Aβ_40_ monomer as the target. Cysteine, which was previously joined with the N-terminus of Aβ_1_ and Aβ_40_, was then covalently cross-linked to the sulfhydryl-functionalized agarose beads using generated disulfide bonds. Eight rounds of screening resulted in the identification of 18 RNA aptamers [80]. To prevent Aβ accumulation, aptamers should stick to the monomeric and oligomeric forms of Aβ. As a result, Aβ oligomer-targeting aptamers must be obtained. Aβ_42_ is more likely to accumulate and be deposed as a significant target for AD because it has a stronger potential to assemble in hazardous species than Aβ_40_ [81]. Aβ_40_ monomers and aggregates have already been quantified using RNA aptamers, suggesting that Aβ aggregation may be inhibited. Aβ and RNA aptamer’s lower binding affinity necessitates improving the inhibition’s specificity and efficacy. Additionally, the majority of RNA aptamers are unstable due to longer sequences and are vulnerable to contamination, which restricts their employment in complex biological organ systems. Different RNA aptamers are included in Table 3 along with their corresponding targets and binding affinities.

##### DNA Aptamers

The focus of research is on DNA aptamers with modest chemical production expenses and superior stability characteristics to enhance the selectivity and application of aptamers in complex systems. The aptamer T-SO508 has been the most frequently used identification molecule in the recognition of Aβ_40_ oligomers [85]. The high tendency of an Aβ monomer to form Aβ oligomers in solutions, as well as the structural significance of an Aβ monomer and an Aβ oligomer, make it difficult to discern between them. Chakravarthy et al. used magnetic bead-assisted SELEX to screen the DNA aptamer RNV95 that targets Aβ_40_ oligomers with low molecular weight. According to structural predictions, RNV95 has a stable stem-loop structure (39-base). In addition, RNV95’s recognition capability in intricate biological systems was tested by identifying that tetrameric/pentameric low molecular weight Aβ accumulates in post-mortem hippocampal tissue. RNV95 can be utilized to identify Aβ oligomers in a variety of affinity assays, according to the findings [86]. T-SO508 and RNV95 are DNA aptamers that are selective of the Aβ oligomer, indicating that the G-quadruplex type of DNA aptamer preferentially binds to Aβ oligomer’s β-structures. They are preferable for building a sensing platform. The DNA aptamers’ base sequence is shorter than the RNA aptamers’, making them easier to synthesize and design (Table 4). The increased efficiency between Aβ and the DNA aptamer might help to enhance the detection sensitivity and inhibition affinity in the clinical diagnosis and therapy of AD [87].

##### Peptide Aptamers

Immunohistochemical labelling and Western blotting on the brains of transgenic AD mice showed that the two peptide aptamers (c-abp2 and n-abp4) exhibited excellent binding efficiencies of 217.97 ± 27.01 pM and 35.80 ± 18.22 pM, respectively, and that they specifically target the Aβ_42_ oligomer. The fundamental examination of the aptamer–Aβ interaction mechanisms also revealed that it could be employed as an excellent instrument to define the impacts of an electric field on the Aβ_40_ monomer and Aβ_40_ accumulation at the single-molecule level. As a result, studying the interaction mechanism between aptamers and Aβ will be advantageous to aptamers’ use in AD treatment [88].

#### 2.7.3. Liposomes

Liposomes are made up of one or more phospholipid bilayers as sphere-shaped vesicles. They are now valuable resources, reagents, and tools in a variety of scientific fields, including mathematics, theoretical physics, biophysics, chemistry, colloid science, biochemistry and biology. Liposomes have since made their way to the market. Liposomes are brilliant new drug delivery methods that characterize an advanced technology to transfer active compounds to the site of action, and numerous formulations are currently in clinical use [89]. Liposomes were first identified in the 1960s and are noteworthy for their non-immunogenicity, flexibility, less toxicity, biodegradability, as well as biocompatibility [90].

Mourtas et al. created polyfunctional liposomes in 2004, which contained a curcumin derivative and a BBB transport mediator anti-transferrin antibody (TrF). Liposomes holding the curcumin derivative or the curcumin derivative plus anti-TrF, demonstrated a great affinity for amyloid plaques, according to the post-mortem brain samples of AD patients. Curcumin-derived liposomes neither inhibit Aβ aggregation nor suppress Aβ deposit staining, as per the authors [91].

#### 2.7.4. Lipid Nanoparticles

Lipid nanoparticles (LNPs) have emerged as appealing carriers for delivering a range of therapeutic compounds in the pharmaceutical sector. LNPs have also been used in various disciplines such as medical imaging, cosmetics, nutrition, agriculture, and other cutting-edge technologies such as nanoreactors. Liposomes, an early form of LNPs, are a very adaptable nanocarrier platform because they can transport both hydrophobic and hydrophilic molecules, such as tiny molecules, proteins, and nucleic acids. Lipid nanoparticles (LNPs), such as solid lipid nanoparticles (SLNs) and nanostructured lipid carriers, were developed to address limitations in colloidal carriers, such as emulsions and liposomes, and polymeric nanoparticles, such as rapid release via the reticuloendothelial system, cell interactions and transfer through the intermembrane, lowered encapsulation efficacy, bad stability, and a shorter shelf life. Targeted drug delivery, enhanced physical stability, and a favorable drug excretion profile have all been made possible by LNPs [92]. In 2012, Bernardi and co-investigator developed lipid-core nanocapsules loaded with indomethacin (IndOH-LNCs) and then examined their capacity to protect cells from neuroinflammation and Aβ1-42-induced cell damage. They discovered that IndOH-LNCs reduced A-induced cell death and suppressed Aβ1-42-induced neuroinflammation in organotypic hippocampus preparations. They also discovered that IndOH-LNC treatment enhanced interleukin-10 production while lowering c-Jun N-terminal kinase phosphorylation [93].

##### Lipoprotein-Based Nanoparticles

Lipoproteins (LPs) are heterogeneous nanoparticles that circulate in the blood stream and are created by the liver and intestines. LPs play an important role in the transfer of dietary and endogenous lipids to target cells via cell membrane receptors or LP lipase on the cell surface. Lipoprotein-based nanoparticles are employed for both therapeutic and diagnostic applications, and they are known to have a great affinity for Aβ, allowing them to be degraded more quickly. For the elimination of Aβ, in 2014, Song and coinvestigators developed a nanoparticle system including apolipoprotein E3–reconstituted high-density lipoprotein (ApoE3–rHDL) 1 h after IV delivery, about 0.4 percent IDg^−1^ (injected dose per gram) of ApoE3–rHDL accessed the mouse brain, and after a month of daily treatment, neurological abnormalities, microgliosis, Aβ deposits, and memory problems were reduced, suggesting that ApoE3–rHDL could be used therapeutically in AD. Its toxicity, however, has yet to be identified [94]. In 2012, Muntimadugu et al. used solid lipid NPs or TFB-loaded poly(lactideco-glycolide) NPs (TFB-NPS) to study the delivery of tarenflurbil (TFB) to the brain through the intranasal route (TFB-SLNs) [95]. TFB is the enantiomer of the NSAID flurbiprofen. TFB is thought to work by reducing Aβ levels through modulating the enzyme c-secretase, which is responsible for the APP cleavage. 

#### 2.7.5. Nanoparticle–Biomolecule Conjugates

A nanoparticle–biomolecule conjugate has biomolecules on its surface. Nanoparticles are nanometer-sized particles, employed in nanobiotechnology to investigate the functionalities of biomolecules. In 2014, Zhang et al. formulated a spherical, dual-function drug delivery device containing TQNP/H102, a β-sheet breaker peptide. QSH- and TGN-targeting peptides were coupled to the nanoparticles’ surfaces to allow Aβ_42_ targeting and BBB transit respectively. This type of technology opens the door to a very specific form of AD treatment [96].

##### Curcumin-Loaded Nanoparticles

Curcumin has been researched and suggested to possess a broad spectrum of biological activities in recent decades because it has a wide range of bioactivity, including anti-AD properties, including neuroprotective potential. Despite these advantages, curcumin does not penetrate the biological system, has low bioavailability, and is susceptible to oxidation and biodegradation. Therefore, to enhance BBB crossing, this barrier can be removed by putting it inside nanocapsules [97]. In 2012, Brambilla et al. created a batch of high molecular weight glycolated polyethylene nanocarriers associated with Aβ_1-42_ that could change conformation in serum, as shown in silico and through modelling [98]. It has also been suggested that they could be applied as an MRI contrast agent to detect Aβ plaque [99].

##### Antibody-Tethered Nanoparticle

Antibodies are responsible for the neutralization of antigens and the stimulation of the complement system. Antibody conjugation to nanoparticles, which are nano-sized biological products, was designed to minimize immune reactions and is now widely employed in diagnosis and research [100]. In 2018, Tamba and coinvestigators investigated the ability of glucose (Glu) and glucose-poly(ethylene glycol) methyl ether amine (Glu-PEG)-coated fluorescent silica NP derivatives (Ru@SNPs) to cross the mouse BBB. Surprisingly, silica NP derivatives were found to easily cross the BBB and operate as good drug-delivery moderators, reaching into the brain via both specific and non-specific processes [101].

#### 2.7.6. Optical Imaging

Optical imaging is a technique that employs light and the unique characteristics of photons to create detailed pictures of organs, tissues, cells, and even molecules. The procedures provide non-invasive or minimally invasive means for viewing within the body. To research neurological changes caused by stopped blood flow, optical imaging should be combined with electroencephalography (EEG). Optical imaging is a relatively new technology that has been employed in molecular biomarker imaging for the identification of AD. There have also been studies of optical imaging for medical purposes [97].

#### 2.7.7. Cubosomes

Cubosomes are very stable nanoparticles generated by the lipid cubic phase and stabilized by an outer corona made of polymers. A single lipid bilayer generates a continuous periodic membrane lattice structure with holes generated by two interlaced water channels in lipid cubic phases. These are biocompatible carriers that are liquid crystalline nanostructured particles. The bioactive substances and proteins come in contact with a 3-D organized bicontinuous curved lipid bilayer disassociated by two water channels. They have the ability to encapsulate amphiphilic, hydrophilic, and hydrophobic molecules, retain regulated drug release and bio-adhesion and are thermodynamically stable, making them ideal carriers for a variety of drug administration routes [102]. In 2015, Elnaggar et al. developed piperine-loaded Tween-integrated monoolein cubosomes (T-cubs) and examined their potency to target AD in the brain [103].

#### 2.7.8. Magnetic Nanoparticles

Magnetic nanoparticles are nanoparticles that can be controlled by magnetic fields. Such particles typically have two components: a magnetic material, generally iron, nickel, or cobalt, and a chemical component with functionality. While nanoparticles have a diameter of less than one micrometer (usually 1–100 nanometers), microbeads have a diameter of 0.5–500 μm. Magnetic nanobeads with a diameter of 50–200 nanometers are magnetic nanoparticle clusters formed of a number of individual magnetic nanoparticles. In 2016, Do et al. developed a magnetic-containing drug-guiding electromagnetic actuator [104]. When exposed to electromagnetic fields of 28 mT (0.43 T/m) or 79.8 mT (1.39 T/m) externally, magnetic particles were observed to pass the BBB. A pulsed magnetic field was also discovered to greatly improve the uptake and transportation speed of magnetic NPs (MNPs) in the brain. MNPs are intriguing possibilities for a variety of biomedical functions. They are made up of a magnetic core, maghemite, and a biocompatible covering, such as polyethylene glycol (PEG). MNPs become more intriguing and useful when functionalized; that is, when they are combined with biological vectors, luminous labels, antibodies, medicines, and so forth. These ideal MNPs are harmless to cells/tissues and stable over long periods of storage. Targeted MNPs have been utilized to identify Aβ plaques in Alzheimer’s disease. Poduslo et al., for example, used a gadolinium-loaded molecular probe to target Aβ plaques in Alzheimer’s disease. Following intravenous administration, their smart system was able to penetrate the BBB and precisely attach to the plaques, which were visualized in MRI. According to their findings, they obtained a more than 9-fold improvement in the cortical and hippocampal areas of AD-transgenic mice [105]. Such devices not only offer enormous promise for early detection but they may also be utilized to directly monitor the success of anti-amyloid therapy. So, these functionalized MNPs are being studied not only for Aβ detection and noninvasive long-term investigations of therapy response but also as viable drug delivery methods [106].

#### 2.7.9. Inorganic Nanoparticles

Investigative work has also been conducted on the potential for inorganic nanoparticles (quantum dots), such as silicon, carbon, indium, cadmium, silver, and graphene, to cross the BBB. Silicon quantum dots are being studied for therapeutic and diagnostic uses since silicon is regarded as biocompatible [107]. Graphene quantum dots (GQDs), a form of semiconducting nanoparticle, are excellent inhibitors of Aβ peptide aggregation. GQDs’ tiny size allows them to easily cross through the blood–brain barrier. Furthermore, GQDs exhibit fluorescent features that can be exploited to monitor Aβ concentrations in vivo. In recent research, the low cytotoxicity and good biocompatibility of GQDs have given them an edge in the applicability and clinical studies for AD when compared to alternative carbon materials. Smaller quantum dots have a broader band gap and a shorter wavelength, whereas bigger quantum dots have a narrower band gap and a longer wavelength. The chemical and physical characteristics of QDs are entirely determined by their size. Fluorescence emission may be adjusted from the near-ultraviolet to the visible and near-infrared spectrums by modifying the QD size and chemical composition, encompassing a wavelength range of 400–2000 nm [108].

##### Nanocomposites

Nanocomposites are solid materials that have many phases, one of which contains nanoscale structures of various dimensions. Stem cell therapy, cancer therapy, antibacterial activities, enzyme immobilization, drug delivery and biosensors are only a few of its applications [109]. In 2018, Chen et al. created a methylene blue (MB; a tau accumulation blocker)-loaded nanocomposite (CeNC/IONC/MSN-T807) that binds tightly to hyperphosphorylated tau. The tau pathway is linked to the clinical development of Alzheimer’s disease symptoms and might be a possible therapeutic target created by an MB-loaded nanocomposite that not only binds to hyperphosphorylated tau but also inhibits many critical pathways of tau-associated AD pathogenesis. They reported that these nanocomposites can alleviate AD symptoms in vitro and in vivo by reducing mitochondrial oxidative stress, inhibiting tau hyperphosphorylation, and preventing neuronal mortality. Treatment with MB-loaded CeNC/IONC/MSN-T807 dramatically improves the memory impairments of AD rats. Our findings suggest that hyperphosphorylated tau-targeted multifunctional nanocomposites might be a viable AD treatment option [110].

#### 2.7.10. Dendrimers—Macromolecular Drug Carriers

Dendrimers are branched as macromolecular structures that are nanoscale in size. They are made up of three structural parts: (a) The central core; (b) The interior dendritic section; and (c) The outer functionalized surface [111]. Studies have shown that dendrimers can help in the solubilization of sparingly soluble medicines in aqueous solutions and are used as drug vehicles for AD. Poly(amidoamine) (PAMAM) dimers operate as modulators of amyloid fibrillar production, improving biocompatibility and synaptic breakdown against Ab oligomers while combating memory loss in AD. Aso et al. recently designed a new type of dendrimer (G4HisMal) with a poly(propylene imine) core and a maltose–histidine shell to shield against AD-induced memory loss in transgenic mice, increase BBB permeability, and enhance biocompatibility. In their transgenic mouse model of AD, G4HisMal substantially increased biocompatibility and BBB penetration, preserved synapses, and avoided mental deterioration [112]. 

#### 2.7.11. Nanoemulsions—Binary Drug Vehicular Systems

Nanoemulsions are nanosized emulsions used to improve the delivery of active medicinal substances. This is a thermodynamically stable isotropic system in which two immiscible liquids are combined to produce a single phase using an emulsifying agent, such as a surfactant and a co-surfactant. Gamma-tocopherol, caffeine, plasmid DNA, aspirin, methyl salicylate, insulin, and nimesulide are some medications that use nanoemulsions for transdermal drug delivery. Sood et al. created a curcumin-loaded nanoemulsion that was stabilized by a surfactant–cosurfactant combination. Curcumin’s water solubility was found to be greatly improved by the formulation [113]. Ferreira et al. examined a nanoemulsion that contained ketoprofen (stabilized with pullulan). Experiments based on an in vitro release revealed a fast (<5 h) release pattern with dramatically better bioavailability, which boosted brain permeability [114]. Memantine, which was originally used to treat influenza, is a non-competitive NMDA receptor antagonist. It affects the glutamatergic system, blocks NMDA receptors, and prevents glutamate from overstimulating these receptors. These produced nanoemulsions also exhibit antioxidant activity, which may be helpful for the treatment of AD, which is characterized by increased free radical formation. With 93.83% cell viability, NEs are low in toxicity and biocompatible. To study the drug’s distribution pattern in rats, the formulation was radiolabeled and delivered by intranasal, oral and intravenous routes. Rat gamma pictures clearly show drug build-up in the brain after intranasal administration. When compared to intravenous treatment, intranasal administration resulted in the greatest absorption of radiolabeled formulation in the brain. It was reported that a nanoemulsion containing memantine might be utilized for intranasal delivery to improve its efficacy against AD [115].

### 2.8. Limitation of Existing Routes for AD Treatment

AD has a complex pathophysiology, and it is currently thought that more effective treatments may be available, employing medications that target numerous molecular pathways and disease-modifying medicines [116]. The majority of the current therapies for AD include taking tablets or capsules orally. However, due to the medications’ limited access to the brain, severe first-pass metabolism, shortened half-life, and potential for side effects when they reach peripheral tissues that are not their intended targets, the systemic distribution of pharmaceuticals to the CNS is a considerable problem [117]. Numerous laboratory and clinical investigations have demonstrated that NPs are a valuable diagnostic and clinical tool. The structure of NPs may be modified, allowing them to be tailored to a specific job, illness, or organ, overcoming many of the issues that plague conventional medications, such as solubility and stability. This method allows for the combination of macromolecules, medicines and imaging agents, resulting in functional NPs that aid in drug administration and diagnostics [118]. There are significant limitations that need the administration of very toxic dosages of liposomes in order to achieve therapeutic effectiveness, including binding to serum proteins and the nonspecific absorption of cationic liposomes by peripheral tissues [119]. The neuroprotective benefits of n-3 PUFA supplementation in AD patients are now being investigated in a number of clinical studies. Despite all of these trials, there has not been a steady improvement in cognitive or neural function in AD patients. Additionally, the effects are often transient. Numerous variables, such as the usage of DHA solely in late-stage AD, inadequate age-influenced bioavailability, or the formulation of DHA, may contribute to these disparities [120].

## 3. Methodology

Most of the articles were selected from PubMed and others were from Google scholar, by searching relevant terms, such as neurological disorder, AD, treatment approaches for AD, nanotechnology for treatment of AD, and other neurological disorders. We preferred the latest published data/articles from every publication. We initiated our search in June 2021, maintained it till August 2022, and we modified the results. After referring to multiple articles, databases and books, we analyzed and summarized the information in this review.

## 4. Conclusions

In the arena of global development, everyone is racing towards the betterment of themselves, which leads to various chronic diseases, as well as neurodegeneration disorders like AD. Developing the appropriate medications is a challenging task for researchers because of the complicated etiology of Alzheimer’s disease. Despite the fact that a number of strategies have been tried to treat AD, including drug repurposing and classic as well as new drug delivery methods, transdermal techniques are also frequently used because of their direct systemic circulation penetration. Bypassing the BBB, which is the main obstacle to treating AD, can be addressed using nanotechnology. Nanoparticles, such as polymeric nanoparticles, aptamers, liposomes, SLN, cubosomes, nanoemulsions, dendrimers, etc., can reach the target areas because of their tiny size. Before AD nanomedicine can be applied in clinical settings, several obstacles must be overcome. Among these, the general and most significant impediment is low targeting efficiency, which may impair the therapeutic impact and cause harm to other organs. Another problem is the dispersion of nanomaterials in the brain. Sequentially targeted nanomaterials merit consideration for precision-targeting in the brain. To avoid diffusion throughout the brain, they must target not just the BBB but also the lesion site. Furthermore, the long-term toxicity of nanomaterials, particularly inorganic nanomaterials, should not be neglected. To ensure that the consumption of these medicines does not accidentally accelerate the onset of amyloid-related diseases, it is imperative to have a complete understanding of how they interact with Aβ and other amyloidogenic proteins. When contemplating the financial aspects of nanoparticles, the production cost is heavier than that of other traditional formulations, which is a significant hindrance to the advancement of nanotechnology.

## Figures and Tables

**Table 1:** Repurposed drugs with their established activity, showing anti-AD activity.

Drug	Present Activity	Mol. wt. (g/mol)	LogP	Anti-Alzheimer’s Effect
Citalopram	Antidepressant	324.39	3.76	Lowers the levels of Aβ in brain interstitial fluid [22]
Deprenyl	Anti-Parkinson	187.28	3.24	APP cleavage assistance [23]
Exendin–4	Antidiabetic	4186.66	3.58	Improves cognitive function and increases synaptic plasticity [24]
Ibuprofen	Anti-inflammatory	206.3	3.97	Alters APP processing, neuroprotective effects [25]
Isradipine	Antihypertensive	371.38	4.28	Neuroprotective effects [26]
Naproxen	Anti-inflammatory	230.26	3.29	Neuroprotective action [27]
Nilvadipine	Antihypertensive	385.4	2.97	Decreases neurotoxicity and Aβ burden [28]
Paroxetine	Antidepressant	329.37	3.1	Reduces Aβ and tau accumulation [29]

**Table 2:** Properties of drugs with conventional oral-based delivery.

Drug	LogP	Clearance	Half Life	Adverse Effect	Dosage Form
Donepezil	4.14	10.5 ± 2 L/h	80 h	Drowsiness, weakness, trouble sleeping, tremor, muscle cramps	Tablet
Rivastigmine	2.45	2.5 ± 0.2 L/h	1.5 h	Stomach pain, loss of appetite, weight loss	Patch and capsule
Galantamine	1.16	18 L/h	7 h	Intravenous atropine, hallucinations	Tablet and capsule
Memantine	3.3	1.2 L/h	60–80 h	Swelling of face, arrhythmia, weight gain	Tablet

**Table 3:** RNA aptamers with respective target and binding affinity.

Name	Target	Binding Affinity (Kd)	Reference
Β55	Aβ_40_ fibril	29 nM	[80]
E2, N2	Aβ_40_ monomer	10.9 µM; 21.6 µM	[82]
KM41, KM33	Aβ_40_ fibril	-	[83]
AbD43, E22P	Aβ_42_ dimer	20 ± 6.0 nM	[84]

**Table 4:** DNA aptamers with respective target and binding affinity.

Name	Target	Binding Affinity (Kd)	Reference
T-SO508	Aβ_40_ oligomer	25 nM	[85]
RNV95	Aβ_40_ oligomer	50–400 nM	[86]
Aβ-79-1H1	Aβ_42_ monomer	63.4 nM	[87]

## Data Availability

Not applicable.

## Abbreviations

AD, Alzheimer’s disease; PD, Parkinson’s disease; BBB, blood–brain barrier; Aβ, amyloid beta; AChE, acetylcholinesterase; BuChE, butyrylcholinesterase; ChEIs, cholinesterase inhibitors; USFDA, United State Food and Drug Administration; TNF-α, tissue necrosis factor-alpha; APP, amyloid protein precursor; NMDA, N-methyl-D-aspartate; GQD, graphene quantum dots; PAMAM, polyamidoamine.

## References

[1] Hider R.C., Ma Y., Molina-Holgado F., Gaeta A., Roy S. (2008). Iron chelation as a potential therapy for neurodegenerative disease. Biochem. Soc. Trans..

[2] Dementia. https://www.who.int/news-room/fact-sheets/detail/dementia.

[3] Feigin V.L., Nichols E., Alam T., Bannick M.S., Beghi E., Blake N., Culpepper W.J., Dorsey E.R., Elbaz A., Ellenbogen R.G. (2019). Global, regional, and national burden of neurological disorders, 1990–2016: A systematic analysis for the Global Burden of Disease Study 2016. Lancet Neurol..

[4] Chan A.L.F., Chien Y.W., Jin Lin S. (2008). Transdermal Delivery of Treatment for Alzheimer’s Disease: Development, Clinical Performance and Future Prospects. Drugs Aging.

[5] Ju Y., Tam K. (2022). Pathological mechanisms and therapeutic strategies for Alzheimer’s disease. Neural Regen. Res..

[6] Hossain M., Jhee S.S., Shiovitz T., McDonald C., Sedek G., Pommier F., Cutler N.R. (2002). Estimation of the Absolute Bioavailability of Rivastigmine in Patients with Mild to Moderate Dementia of the Alzheimer’s Type. Clin. Pharmacokinet..

[7] Selkoe D.J., Hardy J. (2016). The amyloid hypothesis of Alzheimer’s disease at 25 years. EMBO Mol. Med..

[8] Haass C., Selkoe D.J. (2007). Soluble protein oligomers in neurodegeneration: Lessons from the Alzheimer’s amyloid β-peptide. Nat. Rev. Mol. Cell Biol..

[9] Wickner R.B., Bezsonov E.E., Son M., Ducatez M., DeWilde M., Edskes H.K. (2018). Anti-Prion Systems in Yeast and Inositol Polyphosphates. Biochemistry.

[10] Wickner R.B., Edskes H.K., Gorkovskiy A., Bezsonov E.E., Stroobant E.E. (2016). Yeast and Fungal Prions: Amyloid-Handling Systems, Amyloid Structure, and Prion Biology. Adv. Genet..

[11] Wilkinson G.F., Pritchard K. (2015). In Vitro Screening for Drug Repositioning. J. Biomol. Screen..

[12] Tobinick E.L. (2009). The value of drug repositioning in the current pharmaceutical market. Drug News Perspect..

[13] Ashburn T.T., Thor K.B. (2004). Drug repositioning: Identifying and developing new uses for existing drugs. Nat. Rev. Drug Discov..

[14] Corbett A., Pickett J., Burns A., Corcoran J., Dunnett S.B., Edison P., Hagan J.J., Holmes C., Jones E., Katona C. (2012). Drug repositioning for Alzheimer’s disease. Nat. Rev. Drug Discov..

[15] Cramer P.E., Cirrito J.R., Wesson D.W., Lee C.Y.D., Karlo J.C., Zinn A.E., Casali B.T., Restivo J.L., Goebel W.D., James M.J. (2012). ApoE-Directed Therapeutics Rapidly Clear β-Amyloid and Reverse Deficits in AD Mouse Models. Science.

[16] Aicardi G. (2013). New Hope from an Old Drug: Fighting Alzheimer’s Disease with the Cancer Drug Bexarotene (Targretin)?. Rejuvenation Res..

[17] Baruch K., Deczkowska A., Rosenzweig N., Tsitsou-Kampeli A., Sharif A.M., Matcovitch-Natan O., Kertser A., David E., Amit I., Schwartz M. (2016). PD-1 immune checkpoint blockade reduces pathology and improves memory in mouse models of Alzheimer’s disease. Nat. Med..

[18] Vostrov A.A., Taheny M.J., Izkhakov N., Quitschke W.W. (2010). A nuclear factor-binding domain in the 5’-untranslated region of the amyloid precursor protein promoter: Implications for the regulation of gene expression. BMC Res. Notes.

[19] Grossi C., Francese S., Casini A., Rosi M.C., Luccarini I., Fiorentini A., Gabbiani C., Messori L., Moneti G., Casamenti F. (2009). Clioquinol Decreases Amyloid-β Burden and Reduces Working Memory Impairment in a Transgenic Mouse Model of Alzheimer’s Disease. J. Alzheimer’s Dis..

[20] De Felice F.G., Vieira M.N.N., Bomfim T.R., Decker H., Velasco P.T., Lambert M.P., Viola K.L., Zhao W.Q., Ferreira S.T., Klein W.L. (2009). Protection of synapses against Alzheimer’s-linked toxins: Insulin signaling prevents the pathogenic binding of Aβ oligomers. Proc. Natl. Acad. Sci. USA.

[21] Rodriguez-Rivera J., Denner L., Dineley K.T. (2011). Rosiglitazone reversal of Tg2576 cognitive deficits is independent of peripheral gluco-regulatory status. Behav. Brain Res..

[22] Sheline Y.I., West T., Yarasheski K., Swarm R., Jasielec M.S., Fisher J.R., Ficker W.D., Yan P., Xiong C., Frederiksen C. (2014). An Antidepressant Decreases CSF Aβ Production in Healthy Individuals and in Transgenic AD Mice. Sci. Transl. Med..

[23] Jenner P. (2004). Preclinical evidence for neuroprotection with monoamine oxidase-B inhibitors in Parkinson’s disease. Neurology.

[24] McClean P.L., Gault V.A., Harriott P., Hölscher C. (2010). Glucagon-like peptide-1 analogues enhance synaptic plasticity in the brain: A link between diabetes and Alzheimer’s disease. Eur. J. Pharmacol..

[25] Breitner J.C., Baker L.D., Montine T.J., Meinert C.L., Lyketsos C.G., Ashe K.H., Brandt J., Craft S., Evans D.E., Green R.C. (2011). Extended results of the Alzheimer’s disease anti-inflammatory prevention trial. Alzheimer’s Dement..

[26] Anekonda T.S., Quinn J.F., Harris C., Frahler K., Wadsworth T.L., Woltjer R.L. (2011). L-type voltage-gated calcium channel blockade with isradipine as a therapeutic strategy for Alzheimer’s disease. Neurobiol. Dis..

[27] in ’t Veld B.A., Ruitenberg A., Hofman A., Launer L.J., van Duijn C.M., Stijnen T., Breteler M.M., Stricker B.H. (2001). Nonsteroidal Antiinflammatory Drugs and the Risk of Alzheimer’s Disease. N. Engl. J. Med..

[28] Paris D., Quadros A., Humphrey J., Patel N., Crescentini R., Crawford F., Mullan M. (2004). Nilvadipine antagonizes both Aβ vasoactivity in isolated arteries, and the reduced cerebral blood flow in APPsw transgenic mice. Brain Res..

[29] Nelson R., Guo Z., Halagappa V., Pearson M., Gray A., Matsuoka Y., Brown M., Martin B., Iyun T., Maudsley S. (2007). Prophylactic treatment with paroxetine ameliorates behavioral deficits and retards the development of amyloid and tau pathologies in 3xTgAD mice. Exp. Neurol..

[30] Di Stefano A., Iannitelli A., Laserra S., Sozio P. (2011). Drug delivery strategies for Alzheimer’s disease treatment. Expert Opin. Drug Deliv..

[31] Clark C.M., Karlawish J.H.T. (2003). Alzheimer Disease: Current Concepts and Emerging Diagnostic and Therapeutic Strategies. Ann. Intern. Med..

[32] Patocka J., Jun D., Kuca K. (2008). Possible Role of Hydroxylated Metabolites of Tacrine in Drug Toxicity and Therapy of Alzheimers Disease. Curr. Drug Metab..

[33] Danysz W., Parsons C.G. (2003). The NMDA receptor antagonist memantine as a symptomatological and neuroprotective treatment for Alzheimer’s disease: Preclinical evidence. Int. J. Geriat. Psychiatry.

[34] Seltzer B. (2007). Donepezil: An update. Expert Opin. Pharmacother..

[35] Gauthier S. (2001). Cholinergic Adverse Effects of Cholinesterase Inhibitors in Alzheimer’s Disease: Epidemiology and Management. Drugs Aging.

[36] Seltzer B. (2010). Galantamine-ER for the treatment of mild-to-moderate Alzheimer’s disease. Clin. Interv. Aging.

[37] Lee S.H., Kim S.H., Noh Y.H., Choi B.M., Noh G.J., Park W.D., Kim E.J., Cho I.H., Bae C.S. (2016). Pharmacokinetics of Memantine after a Single and Multiple Dose of Oral and Patch Administration in Rats. Basic Clin. Pharmacol. Toxicol..

[38] Abetz L., Rofail D., Mertzanis P., Heelis R., Rosa K., Tellefsen C., de Climens A.R., McBurney C., Thomas S. (2009). Alzheimer’s disease treatment: Assessing caregiver preferences for mode of treatment delivery. Adv. Ther..

[39] Claxton A.J., Cramer J., Pierce C. (2001). A systematic review of the associations between dose regimens and medication compliance. Clin. Ther..

[40] Muramatsu R.S., Litzinger M.H.J., Fisher E., Takeshita J. (2010). Alternative formulations, delivery methods, and administration options for psychotropic medications in elderly patients with behavioral and psychological symptoms of dementia. Am. J. Geriatr. Pharmacother..

[41] Farlow M.R., Salloway S., Tariot P.N., Yardley J., Moline M.L., Wang Q., Brand-Schieber E., Zou H., Hsu T., Satlin A. (2010). Effectiveness and tolerability of high-dose (23 mg/d) versus standard-dose (10 mg/d) donepezil in moderate to severe Alzheimer’s disease: A 24-week, randomized, double-blind study. Clin. Ther..

[42] Yan Y.D., Woo J.S., Kang J.H., Yong C.S., Choi H.G. (2010). Preparation and Evaluation of Taste-Masked Donepezil Hydrochloride Orally Disintegrating Tablets. Biol. Pharm. Bull..

[43] Robinson D.M., Plosker G.L. (2006). Galantamine Extended Release. CNS Drugs.

[44] Brodaty H., Corey-Bloom J., Potocnik F.C.V., Truyen L., Gold M., Damaraju C.R.V. (2005). Galantamine Prolonged-Release Formulation in the Treatment of Mild to Moderate Alzheimer’s Disease. Dement. Geriatr. Cogn. Disord..

[45] Scholz O.A., Wolff A., Schumacher A., Giannola L.I., Campisi G., Ciach T., Velten T. (2008). Drug delivery from the oral cavity: Focus on a novel mechatronic delivery device. Drug Discov. Today.

[46] Whelpton R., Hurst P. (1985). Bioavailability of oral physostigmine. N. Engl. J. Med..

[47] Bolourchian N., Hadidi N., Foroutan S., Shafaghi B. (2009). Development and optimization of a sublingual tablet formulation for physostigmine salicylate. Acta Pharm..

[48] Bassil N., Thaipisuttikul P., Grossberg G.T. (2010). Memantine ER, a once-daily formulation for the treatment of Alzheimer’s disease. Expert Opin. Pharmacother..

[49] Potyk D. (2005). Treatments for Alzheimer disease. South. Med. J..

[50] Imbimbo B.P. (2001). Pharmacodynamic-tolerability relationships of cholinesterase inhibitors for Alzheimer’s disease. CNS Drugs.

[51] Levy D., Glikfeld P., Grunfeld Y., Grunwald J., Kushnir M., Levy A., Meshulam Y., Spiegelstein M., Zehavi D., Fisher A., Fisher A., Hanin I., Lachman C. (1986). A Novel Transdermal Therapeutic System as a Potential Treatment for Alzheimer’s Disease. Alzheimer’s and Parkinson’s Disease.

[52] Walter K., Müller M., Barkworth M.F., Nieciecki A.V., Stanislaus F. (1995). Pharmacokinetics of physostigmine in man following a single application of a transdermal system. Br. J. Clin. Pharmacol..

[53] Guy R.H., Kalia Y.N., Delgado-Charro M.B., Merino V., López A., Marro D. (2000). Iontophoresis: Electrorepulsion and electroosmosis. J. Control. Release.

[54] Upasani R.S., Banga A.K. (2004). Response surface methodology to investigate the iontophoretic delivery of tacrine hydrochloride. Pharm. Res..

[55] Kankkunen T., Sulkava R., Vuorio M., Kontturi K., Hirvonen J. (2002). Transdermal iontophoresis of tacrine in vivo. Pharm. Res..

[56] Jaskari T., Vuorio M., Kontturi K., Urtti A., Manzanares J.A., Hirvonen J. (2000). Controlled transdermal iontophoresis by ion-exchange fiber. J. Control. Release.

[57] Venkatraman S., Gale R. (1998). Skin adhesives and skin adhesion. 1. Transdermal drug delivery systems. Biomaterials.

[58] Nguyen T.T., Giau V.V., Vo T.K. (2017). Current advances in transdermal delivery of drugs for Alzheimer’s disease. Indian J. Pharmacol..

[59] Tezel A., Sens A., Mitragotri S. (2002). A theoretical analysis of low-frequency sonophoresis: Dependence of transdermal transport pathways on frequency and energy density. Pharm. Res..

[60] Khanmohammadi M., Elmizadeh H., Ghasemi K. (2015). Investigation of Size and Morphology of Chitosan Nanoparticles Used in Drug Delivery System Employing Chemometric Technique. Iran. J. Pharm. Res..

[61] Heydorn W.E. (1997). Donepezil (E2020): A new acetylcholinesterase inhibitor. Review of its pharmacology, pharmacokinetics, and utility in the treatment of Alzheimer’s disease. Expert Opin. Investig. Drugs.

[62] Valia K.H., Ramaraju V.S. (2008). Transdermal Methods and Systems for Treating Alzheimer’s Disease. U.S. Patent.

[63] Kim K.H., Gwak H.S. (2011). Effects of vehicles on the percutaneous absorption of donepezil hydrochloride across the excised hairless mouse skin. Drug Dev. Ind. Pharm..

[64] Saluja S., Kasha P.C., Paturi J., Anderson C., Morris R., Banga A.K. (2013). A novel electronic skin patch for delivery and pharmacokinetic evaluation of donepezil following transdermal iontophoresis. Int. J. Pharm..

[65] Small G., Dubois B. (2007). A review of compliance to treatment in Alzheimer’s disease: Potential benefits of a transdermal patch. Curr. Med. Res. Opin..

[66] Prvulovic D., Hampel H., Pantel J. (2010). Galantamine for Alzheimer’s disease. Expert Opin. Drug Metab. Toxicol..

[67] Park C.W., Son D.D., Kim J.Y., Oh T.O., Ha J.M., Rhee Y.S., Park E.S. (2012). Investigation of formulation factors affecting in vitro and in vivo characteristics of a galantamine transdermal system. Int. J. Pharm..

[68] Woo F.Y., Basri M., Masoumi H.R.F., Ahmad M.B., Ismail M. (2015). Formulation optimization of galantamine hydrobromide loaded gel drug reservoirs in transdermal patch for Alzheimer’s disease. Int. J. Nanomed..

[69] McKeage K. (2010). Spotlight on memantine in moderate to severe Alzheimer’s disease. Drugs Aging.

[70] Del Rio-Sancho S., Serna-Jiménez C.E., Calatayud-Pascual M.A., Balaguer-Fernández C., Femenía-Font A., Merino V., López-Castellano A. (2012). Transdermal absorption of memantin—Effect of chemical enhancers, iontophoresis, and role of enhancer lipophilicity. Eur. J. Pharm. Biopharm..

[71] Wang J.M., Singh C., Liu L., Irwin R.W., Chen S., Chung E.J., Thompson R.F., Brinton R.D. (2010). Allopregnanolone reverses neurogenic and cognitive deficits in mouse model of Alzheimer’s disease. Proc. Natl. Acad. Sci. USA.

[72] Lipinski C.A. (2000). Drug-like properties and the causes of poor solubility and poor permeability. J. Pharm. Toxicol. Methods.

[73] Irwin R.W., Solinsky C.M., Loya C.M., Salituro F.G., Rodgers K.E., Bauer G., Rogawski M.A., Brinton R.D. (2015). Allopregnanolone preclinical acute pharmacokinetic and pharmacodynamic studies to predict tolerability and efficacy for Alzheimer’s disease. PLoS ONE.

[74] Singh S., Singh M., Gambhir I.S. (2008). Nanotechnology for Alzheimer’s disease detection. Dig. J. Nanomater. Biosyst..

[75] Vestergaard M., Kerman K., Kim D.K., Ha M.H., Tamiya E. (2008). Detection of Alzheimer’s tau protein using localised surface plasmon resonance-based immunochip. Talanta.

[76] Sun D., Li N., Zhang W., Zhao Z., Mou Z., Huang D., Liu J., Wang W. (2016). Design of PLGA-functionalized quercetin nanoparticles for potential use in Alzheimer’s disease. Colloids Surf. B Biointerfaces.

[77] Huo X., Zhang Y., Jin X., Li Y., Zhang L. (2019). A novel synthesis of selenium nanoparticles encapsulated PLGA nanospheres with curcumin molecules for the inhibition of amyloid β aggregation in Alzheimer’s disease. J. Photochem. Photobiol. B Biol..

[78] Vilella A., Belletti D., Sauer A.K., Hagmeyer S., Sarowar T., Masoni M., Stasiak N., Mulvihill J.J.E., Ruozi B., Forni F. (2018). Reduced plaque size and inflammation in the APP23 mouse model for Alzheimer’s disease after chronic application of polymeric nanoparticles for CNS targeted zinc delivery. J. Trace Elem. Med. Biol..

[79] Tseng Y.T., Harroun S.G., Wu C.W., Mao J.Y., Chang H.T., Huang C.C. (2017). Satellite-like Gold Nanocomposites for Targeted Mass Spectrometry Imaging of Tumor Tissues. Nanotheranostics.

[80] Ylera F., Lurz R., Erdmann V.A., Fürste J.P. (2002). Selection of RNA Aptamers to the Alzheimer’s Disease Amyloid Peptide. Biochem. Biophys. Res. Commun..

[81] Findeis M.A. (2007). The role of amyloid β peptide 42 in Alzheimer’s disease. Pharmacol. Ther..

[82] Takahashi T., Tada K., Mihara H. (2009). RNA aptamers selected against amyloid β-peptide (Aβ) inhibit the aggregation of Aβ. Mol. BioSyst..

[83] Rahimi F., Murakami K., Summers J.L., Chen C.H.B., Bitan G. (2009). RNA aptamers generated against oligomeric Abeta40 recognize common amyloid aptatopes with low specificity but high sensitivity. PLoS ONE.

[84] Murakami K., Obata Y., Sekikawa A., Ueda H., Izuo N., Awano T., Takabe K., Shimizu T., Irie K. (2020). An RNA aptamer with potent affinity for a toxic dimer of amyloid β42 has potential utility for histochemical studies of Alzheimer’s disease. J. Biol. Chem..

[85] Tsukakoshi K., Abe K., Sode K., Ikebukuro K. (2012). Selection of DNA Aptamers That Recognize α-Synuclein Oligomers Using a Competitive Screening Method. Anal. Chem..

[86] Chakravarthy M., AlShamaileh H., Huang H., Tannenberg R.K., Chen S., Worrall S., Dodd P.R., Veedu R.N. (2018). Development of DNA aptamers targeting low-molecular-weight amyloid-β peptide aggregates in vitro. Chem. Commun..

[87] Zheng Y., Zhang L., Zhao J., Li L., Wang M., Gao P., Wang Q., Zhang X., Wang W. (2022). Advances in aptamers against Aβ and applications in Aβ detection and regulation for Alzheimer’s disease. Theranostics.

[88] Zheng Y., Wang Q., Yang X., Nie W., Zou L., Liu X., Wang K. (2019). Aptamer as a Tool for Investigating the Effects of Electric Field on Aβ _40_ Monomer and Aggregates Using Single-Molecule Force Spectroscopy. Anal. Chem..

[89] Akbarzadeh A., Rezaei-Sadabady R., Davaran S., Joo S.W., Zarghami N., Hanifehpour Y., Samiei M., Kouhi M., Nejati-Koshki K. (2013). Liposome: Classification, preparation, and applications. Nanoscale Res. Lett..

[90] Bozzuto G., Molinari A. (2015). Liposomes as nanomedical devices. Int. J. Nanomed..

[91] Mourtas S., Lazar A.N., Markoutsa E., Duyckaerts C., Antimisiaris S.G. (2014). Multifunctional nanoliposomes with curcumin–lipid derivative and brain targeting functionality with potential applications for Alzheimer disease. Eur. J. Med. Chem..

[92] Naseri N., Valizadeh H., Zakeri-Milani P. (2015). Solid Lipid Nanoparticles and Nanostructured Lipid Carriers: Structure, Preparation and Application. Adv. Pharm. Bull..

[93] Bernardi A., Frozza R.L., Meneghetti A., Hoppe J.B., Battastini A.M.O., Pohlmann A.R., Guterres S.S., Salbego C.G. (2012). Indomethacin-loaded lipid-core nanocapsules reduce the damage triggered by Aβ1-42 in Alzheimer’s disease models. Int. J. Nanomed..

[94] Song Q., Huang M., Yao L., Wang X., Gu X., Chen J., Chen J., Huang J., Hu Q., Kang T. (2014). Lipoprotein-Based Nanoparticles Rescue the Memory Loss of Mice with Alzheimer’s Disease by Accelerating the Clearance of Amyloid-Beta. ACS Nano.

[95] Muntimadugu E., Dhommati R., Jain A., Challa V.G.S., Shaheen M., Khan W. (2016). Intranasal delivery of nanoparticle encapsulated tarenflurbil: A potential brain targeting strategy for Alzheimer’s disease. Eur. J. Pharm. Sci..

[96] Zhang C., Zheng X., Wan X., Shao X., Liu Q., Zhang Z., Zhang Q. (2014). The potential use of H102 peptide-loaded dual-functional nanoparticles in the treatment of Alzheimer’s disease. J. Control. Release.

[97] Ahmad J., Akhter S., Rizwanullah M., Khan M.A., Pigeon L., Addo R.T., Greig N.H., Midoux P., Pichon C., Kamal M.A. (2017). Nanotechnology Based Theranostic Approaches in Alzheimer’s Disease Management: Current Status and Future Perspective. Curr. Alzheimer Res..

[98] Brambilla D., Verpillot R., Le Droumaguet B., Nicolas J., Taverna M., Kóňa J., Lettiero B., Hashemi S.H., De Kimpe L., Canovi M. (2012). PEGylated nanoparticles bind to and alter amyloid-beta peptide conformation: Toward engineering of functional nanomedicines for Alzheimer’s disease. ACS Nano.

[99] Patil R., Gangalum P.R., Wagner S., Portilla-Arias J., Ding H., Rekechenetskiy A., Konda B., Inoue S., Black K.L., Ljubimova J.Y. (2015). Curcumin Targeted, Polymalic Acid-Based MRI Contrast Agent for the Detection of Aβ Plaques in Alzheimer’s Disease: Curcumin Targeted, Polymalic Acid-Based MRI Contrast. Macromol. Biosci..

[100] Arruebo M., Valladares M., González-Fernández Á. (2009). Antibody-Conjugated Nanoparticles for Biomedical Applications. J. Nanomater..

[101] Tamba B.I., Streinu V., Foltea G., Neagu A.N., Dodi G., Zlei M., Tijani A., Stefanescu C. (2018). Tailored surface silica nanoparticles for blood-brain barrier penetration: Preparation and in vivo investigation. Arab. J. Chem..

[102] Karami Z., Hamidi M. (2016). Cubosomes: Remarkable drug delivery potential. Drug Discov. Today.

[103] Elnaggar Y., Etman S., Abdelmonsif D., Abdallah O. (2015). Novel piperine-loaded Tween-integrated monoolein cubosomes as brain-targeted oral nanomedicine in Alzheimer’s disease: Pharmaceutical, biological, and&nbsp;toxicological studies. Int. J. Nanomed..

[104] Do T.D., Amin F.U., Noh Y., Kim M.O., Yoon J. (2016). Guidance of Magnetic Nanocontainers for Treating Alzheimer’s Disease Using an Electromagnetic, Targeted Drug-Delivery Actuator. J. Biomed. Nanotechnol..

[105] Poduslo J.F., Wengenack T.M., Curran G.L., Wisniewski T., Sigurdsson E.M., Macura S.I., Borowski B.J., Jack C.R. (2002). Molecular Targeting of Alzheimer’s Amyloid Plaques for Contrast-Enhanced Magnetic Resonance Imaging. Neurobiol. Dis..

[106] Amiri H., Saeidi K., Borhani P., Manafirad A., Ghavami M., Zerbi V. (2013). Alzheimer’s Disease: Pathophysiology and Applications of Magnetic Nanoparticles as MRI Theranostic Agents. ACS Chem. Neurosci..

[107] Sivasankarapillai V.S., Jose J., Shanavas M.S., Marathakam A., Uddin M.d.S., Mathew B. (2019). Silicon Quantum Dots: Promising Theranostic Probes for the Future. Curr. Drug Targets.

[108] Ghosh S., Sachdeva B., Sachdeva P., Chaudhary V., Rani G.M., Sinha J.K. (2022). Graphene quantum dots as a potential diagnostic and therapeutic tool for the management of Alzheimer’s disease. Carbon Lett..

[109] Kamigaito O. (1991). What can be improved by nanometer composites?. J. Jpn. Soc. Powder Powder Metall..

[110] Chen Q., Du Y., Zhang K., Liang Z., Li J., Yu H., Ren R., Feng J., Jin Z., Li F. (2018). Tau-Targeted Multifunctional Nanocomposite for Combinational Therapy of Alzheimer’s Disease. ACS Nano.

[111] Jose J., Charyulu R.N. (2016). Prolonged drug delivery system of an antifungal drug by association with polyamidoamine dendrimers. Int. J. Pharm. Investig..

[112] Aso E., Martinsson I., Appelhans D., Effenberg C., Benseny-Cases N., Cladera J., Gouras G., Ferrer I., Klementieva O. (2019). Poly(propylene imine) dendrimers with histidine-maltose shell as novel type of nanoparticles for synapse and memory protection. Nanomed. Nanotechnol. Biol. Med..

[113] Sood S., Jain K., Gowthamarajan K. (2014). Optimization of curcumin nanoemulsion for intranasal delivery using design of experiment and its toxicity assessment. Colloids Surf. B Biointerfaces.

[114] Ferreira L.M., Cervi V.F., Gehrcke M., da Silveira E.F., Azambuja J.H., Braganhol E., Sari M.H., Zborowski V.A., Nogueira C.W., Cruz L. (2015). Ketoprofen-loaded pomegranate seed oil nanoemulsion stabilized by pullulan: Selective antiglioma formulation for intravenous administration. Colloids Surf. B Biointerfaces.

[115] Kaur A., Nigam K., Srivastava S., Tyagi A., Dang S. (2020). Memantine nanoemulsion: A new approach to treat Alzheimer’s disease. J. Microencapsul..

[116] Castellani R.J., Perry G. (2012). Pathogenesis and Disease-modifying Therapy in Alzheimer’s Disease: The Flat Line of Progress. Arch. Med. Res..

[117] Tonda-Turo C., Origlia N., Mattu C., Accorroni A., Chiono V. (2019). Current Limitations in the Treatment of Parkinson’s and Alzheimer’s Diseases: State-of-the-Art and Future Perspective of Polymeric Carriers. Curr. Med. Chem..

[118] Neganova M.E., Aleksandrova Y.R., Sukocheva O.A., Klochkov S.G. (2022). Benefits and limitations of nanomedicine treatment of brain cancers and age-dependent neurodegenerative disorders. Semin. Cancer Biol..

[119] Agrawal M., Ajazuddin, Tripathi D.K., Saraf S., Saraf S., Antimisiaris S.G., Mourtas S., Hammarlund-Udenaes M., Alexander A. (2017). Recent advancements in liposomes targeting strategies to cross blood-brain barrier (BBB) for the treatment of Alzheimer’s disease. J. Control. Release.

[120] Passeri E., Elkhoury K., Morsink M., Broersen K., Linder M., Tamayol A., Malaplate C., Yen F.T., Arab-Tehrany E. (2022). Alzheimer’s Disease: Treatment Strategies and Their Limitations. Int. J. Mol. Sci..

